# Inclusive finance, industrial structure upgrading and farmers’ income: Empirical analysis based on provincial panel data in China

**DOI:** 10.1371/journal.pone.0258860

**Published:** 2021-10-20

**Authors:** Guibo Liu, Huimin Fang, Xiaoxian Gong, Feifei Wang

**Affiliations:** 1 College of Finance and Statistics, Hunan University, Changsha, Hunan, China; 2 School of Economics, Management and Statistics, University of Bologna, Bologna, Italy; 3 School of Accountancy, Shandong Technology and Business University, Yantai, Shandong, China; 4 Glorious Sun School of Business Management, Donghua University, Shanghai, China; China University of Mining and Technology, CHINA

## Abstract

This paper empirically studies the relationship among inclusive finance, industrial structure upgrading and farmers’ income, using the panel data of 28 provinces in China from 2006 to 2016.The research finds that inclusive finance can significantly promote the increase of farmers’ income. Moreover, the Upgrading of Industry Structure (UIS) is the intermediary mechanism of inclusive finance to promote the increase of farmers’ income, and this intermediary mechanism is heterogeneous among farmers with different income levels. Finally, the promotion effect of the UIS on farmers’ income is affected by the threshold effect of inclusive finance. Compared with the development level of low inclusive finance, the promotion effect of the UIS on farmers’ income is stronger under the development level of high inclusive finance. According to the results of empirical analysis, we suggest that the development strategy of inclusive finance should aim at the industrial development in rural areas, promote the organic connection between farmers and modern agricultural industry, and drive farmers to increase their income through the transformation and upgrading of rural industries.

## Introduction

As a main agricultural country with the largest population and the longest history in the world [1], the continuous increase of farmers’ income is of particularly practical significance for China’s economic development and social stability. The income level of rural residents is still low and the growth rate is relatively slow compared with that of urban residents, although the income of Chinese farmers has increased rapidly in the past few decades. According to the data released by the National Bureau of Statistics of China, the per capita disposable income of rural residents was RMB 133.6 in 1978 and RMB 16,020.7 in 2019, an increase of nearly 120 times. The per capita disposable income of urban residents in the same period was RMB 343.4 in 1978 and RMB 42,358.8 in 2019, an increase of more than 123 times. These figures fully illustrate the severity of the problem of farmers’ income in China. Beyond this, the international economic situation has already been changed and China’s economy has stepped into a new stage. The pressure of sustained growth of farmers’ income has gradually become prominent based on the profound changes of the external environment and internal conditions faced by agricultural development. How to further improve the income level of farmers and promote the sustainable development of rural economy will be the focused questions of the Chinese government and scholars in the future.

Inclusive finance emphasizes the accessibility of useful and affordable financial products and services to individuals and businesses [2]. Farmers are the key customers of inclusive finance, so inclusive finance is an important factor affecting farmers’ income. The Chinese government has included the development of inclusive finance in the national strategy level and has taken a series of measures in the construction of inclusive finance system and input of inclusive finance, so that inclusive finance has achieved rapid development. Taking agriculture-related loans as an example, data released by the Chinese government shows that the balance of agriculture-related loans in China in 2020 is RMB 38.95 trillion, an increase of RMB 3.94 trillion compared with the same period in 2019, and a year-on-year increase of 10.7%. Among them, the balance of bank loans to rural households stood at RMB 11.81 trillion, an increase of RMB 1.51 trillion over the same period in 2019 and an increase of 14.2% year on year. However, the development of inclusive finance has also exposed some problems, which are highlighted in the following aspects. In the first place, the “elite capture” mechanism [3] also exists in the agricultural loan market, and the agricultural loan funds are occupied by a small number of elite farmers, while ordinary farmers with credit needs may not get credit funds, which will restrict the motivation of farmers to increase their income by using credit funds. Secondly, in the process of development, inclusive finance shows strong characteristics of blood transfusion. It is mainly committed to meet the credit availability of vulnerable groups such as farmers, with less support for the economic transformation and upgrading in rural areas. However, the latter is the long-term mechanism to promote the sustainable growth of farmers’ income. Finally, the financial system is committed to pursuing interest compatibility and sustainable development goals [4]. Under the traditional development model, inclusive finance is faced with the problem of high cost and high risk in providing financial services to vulnerable groups such as farmers and small and micro enterprises.

The UIS is the source of power to achieve economic growth [5, 6]. On the one hand, the backward industrial structure and development mode in China’s rural areas have restricted the sustainable development of rural economy and the sustainable growth of farmers’ income, and at the same time, they cannot meet the new requirements of rural productivity under the new situation. The marginal effect of the original blood transfusion inclusive financial development model on farmers’ income has gradually weakened. The development model of inclusive finance should be transformed to give full play to its hematopoietic function and guiding role, and support the UIS. It is not only beneficial to the sustainable development of inclusive finance itself, but also to promote the sustainable growth of farmers’ income. In view of this, this paper construct inclusive financial indicator system to empirically test whether inclusive finance will promote the growth of farmers’ income. On this basis, it analyzes whether the UIS is the realization mechanism of inclusive financial promoting the growth of farmers’ income. And further studies the effect of the UIS on farmers’ income under the influence of the threshold effect of inclusive finance. This is helpful to clarify the relationship among inclusive finance, UIS and farmers’ income, and has certain guiding significance and reference value for effectively playing inclusive finance to promote farmers’ income increase.

The rest of this paper is arranged as follows: the second part is literature review and research hypothesis. The third part is the research design, including sample selection and data sources, inclusive finance measurement, calculation of the UIS, control variables, model setting, descriptive statistics and analysis results of farmers’ income. The fourth part is empirical results and analysis, including unit root test and cointegration test, regression results and analysis of benchmark model, regression results and analysis of mediating effect model, endogeneity problem and robustness test. The fifth part is further study and the last part is the conclusion and implication.

### Literature review and research hypothesis

#### Study on the influence of inclusive finance on farmers’ income

Until now, the financial system has not fully possessed the ideal function of a competitive market and cannot provide fair and open trading opportunities for every economic subject. Financial exclusion refers to the phenomenon that the poor and disadvantaged groups have difficulty in obtaining financial services through formal channels [7]. Agricultural industry has the weak characteristics of long production cycle, high risk and low return rate [8], and thus rural areas have been subjected to severe financial exclusion [9, 10]. Inclusive finance is a financial system that enables all social classes and groups to enjoy extensive and barrierless financial services [11]. The reason of this financial system proposed is to solve the financial exclusion faced by vulnerable groups and promote the inclusive development of finance. Relevant scholars have focused on the relationship between inclusive finance and poverty reduction and income increase of farmers. Most scholars believe that farmers and other vulnerable groups can make use of the credit capital provided by inclusive finance for investment and production operation, thus helping to increase their income level and reduce their poverty [12, 13].

The following is a simple theoretical model to illustrate the relationship between inclusive finance and farmers’ income. This paper refers to the research of Aghion and Howitt (2009) [14], Zheng and Zhu (2019) [15] add rural financial exclusion factors to the original model. It is also assumed that the differences in farmers’ productivity cause the differences in the difficulty of obtaining credit resources. Assuming that there are *N* farmers in the rural economy of the province, and each farmer *i* has *e*_*i*_ unit of capital stock in the period of *t*, then the total capital stock of all farmers in the province is:

$$K_{t}=\sum_{i=1}^{N}e_{i}$$ (1)

Assuming each farmer has diminishing marginal productivity, the production function of each farmer *i* can be expressed as:

$$y_{i}=\tau_{i}k_{i}$$ (2)

Among them, parameter *τ* represents the productivity of each farmer. The productivity of each farmer is different, and its magnitude depends on the initial wealth of farmers, the situation of local economic development and other factors. Moreover, parameter *τ* meets the following requirements:

$$\underset{︸}{\tau_{1}>\tau_{2}\ldots >\tau_{i}>\cdots >\tau_{m-1}>}\tau_{m}\underset{︸}{>\tau_{m+1}>\cdots \tau_{j}>\cdots >\tau_{N}}$$ (3)

In Eq (3), *τ*_*m*_ represents the productivity of marginal producers. The left side of *τ*_*m*_ is the farmer with higher productivity, and the right side of *τ*_*m*_ is the farmer with lower productivity. Every rational farmer *i* will choose the amount of capital used *k*_*i*_ to maximize his return. Therefore, the production behavior of each farmer *i* can be written as:

$$\pi_{i}=\tau_{i}k_{i}-r(k_{i}-e_{i})$$ (4)

In Eq (4), *π*_*i*_ represents the income of the *i*th farmer, and *r* is the market interest rate. This equation also needs to satisfy the credit constraint conditions, as shown in Eq (5):

$$k_{i}\leq ve_{i}$$ (5)

In Eq (5), the value range of *v* is [1,+∞]. The larger the value of *v*, the lower the degree of financial exclusion, that is, the higher the development level of inclusive finance in the province.

The equilibrium of the provincial financial market requires that the total amount of capital used is equal to the total capital stock *K*_*t*_, by setting the equilibrium interest rate equal to the interest rate of marginal producer *τ*_*m*_. At this point, the total capital use will be equal to the capital use of marginal producers plus the maximum amount available to all farmers when *τ*_*i*_ is greater than the market interest rate. The equilibrium equation is (6):

$$k_{m}+v\sum_{0}^{m-1}e_{i}=K_{t}$$ (6)

Since 0≤*k*_*m*_≤*ve*_*m*_, the equilibrium requirements of the rural financial market in the province are shown in Eq (7):

$$\sum_{0}^{m-1}e_{i}\leq \frac{K_{t}}{v}\leq \sum_{0}^{m}e_{i}$$ (7)

With the improvement of the development level of inclusive finance, the equilibrium state of the provincial financial market will change. Farmers with lower productivity than the marginal producers previously constrained by credit will replace the original marginal producers and become the new marginal producers. The total output of the above scenario can be expressed as:

$$Y_{t}=\tau_{m}k_{m}+\sum_{0}^{m-1}\tau_{i}k_{i}=\tau_{m}k_{m}+v\sum_{0}^{m-1}\tau_{m}\;e_{m}$$ (8)

According to Eq (8) and market equilibrium condition (6), we can get:

$$Y_{t}=\tau_{m}K_{t}+v\sum_{0}^{m-1}(\tau_{i}-\tau_{m})e_{i}$$ (9)

Since the farmer’s productivity *τ*_*i*_ > *τ*_*m*_, for all *i* < *m*, we can get:

$$\frac{\partial Y_{t}}{\partial v}=\sum_{0}^{m-1}(\tau_{i}-\tau_{m})e_{i}>0$$ (10)

The above formulas show that, with the improvement of the development level of inclusive finance, some farmers with low initial wealth and low productivity will obtain more credit capital to engage in production activities, thus increasing the income level. Therefore, we can conclude that inclusive finance can promote the increase of farmers’ income.

Based on the above analysis, this paper proposes the following research hypotheses:

H1: Inclusive finance can significantly promote the growth of farmers’ income.

#### Intermediating effect

About the mechanism of inclusive finance to improve income and reduce poverty of rural residents, it can be found that there are mainly two types of micro mechanism and macro mechanism based on the research of relevant scholars. The micro mechanism shows that inclusive finance influences residents’ poverty reduction and income increase through consumption smoothing effect, career choice effect, human capital investment effect and risk management effect [16, 17]. By contrast, the macro mechanism is through economic growth effect, income distribution effect and industrial diffusion effect [18–20]. Further, relevant scholars have focused on how inclusive finance should transform the credit support mechanism, so as to better promote the sustainable development of rural economy and the sustained increase of farmers’ income. Zheng and Zhu (2019) believe that the development strategy of inclusive finance should aim at the economic and industrial development of poverty-stricken counties, combined with the local industrial poverty alleviation and poverty alleviation of production lines [15]. All above strategies together can improve the economic opportunities faced by the poverty-stricken groups, improve the self-development ability of the poverty-stricken groups, and better promote the poverty reduction and income increase of the rural residents. Hu et al. (2021) found through research that inclusive finance can improve the productivity of agricultural total factor and this economic effect of inclusive finance is realized by providing credit support for rural production transformation based on division of labor and collaboration [2].

Is the UIS a mechanism for inclusive finance to promote farmers’ income? Until now, there is no relevant research, but it can be analyzed from the following two aspects. First, the development of finance promotes the UIS. Finance can provide capital support for industrial development to alleviate financing constraints in the process of industrial development. Moreover, finance can realize the efficient flow of capital between industrial sectors by reasonably allocating capital to different industrial sectors. Relevant scholars have studied the promotion effect of financial development on industrial upgrading from different perspectives. From the perspective of the credit creation function of financial institutions, Schumpeter (1934)found that banks could continuously invest funds in the field of innovation activities, so as to achieve the effect of promoting the UIS and economic growth [21]. With the help of relevant theories of information economics, Chava et al. (2013) proposed that the development of the financial system could reduce information asymmetry and transaction costs, thus promoting capital accumulation and technological innovation, and thus driving the UIS and economic development [22]. Second, the UIS promotes economic growth. Rostow (1963) believed that in the evolution process of unbalanced dynamic structure, the constantly transforming industrial structure enhanced the core competence of the industry, optimized the allocation of technology, capital and labor, and finally improved the speed and quality of economic development [23]. Peneder (2002) revealed the core reason why the UIS promotes economic growth [24]. He pointed out that different industrial sectors have differences in productivity and productivity growth rate. When input elements flow from sectors with low productivity level or low productivity growth rate to sectors with high productivity level or high productivity growth rate, the "structural dividend" is generated to promote high economic growth. Thabet (2015) found through empirical analysis that UIS can significantly improve total factor productivity, based on the panel data of 138 Tunisian enterprises from 1998 to 2004 [25]. Inclusive finance can guide financial resources to serve agricultural production and pay attention to the financial needs of small farmers, an important constituent group of agricultural industry [26]. If the above links are established, then we can argue that the UIS is a mechanism for inclusive finance to promote farmers’ income.

Based on the above analysis, this paper proposes the following research hypotheses:

H2: Inclusive finance can increase farmers’ income by promoting UIS, and UIS is the intermediary mechanism through which inclusive finance promotes farmers’ income growth.

### Research design

#### Sample selection and data sources

This paper selects 28 provinces in China as the research object and sets the research sample period as 2006 ~2016. The data is mainly from the EPS global statistical data analysis platform and the Chinese provincial administrative statistical yearbooks. The data of the number of business outlets and the number of employees of banking financial institutions comes from the financial operation reports of each region. For the missing data, this paper used the moving average method to make up the data, and finally obtained 308 panel data observation values after sorting out.

#### Inclusive finance measurement

Since there is no statistical value of inclusive finance index in the database, this paper constructs the indicator system of inclusive finance and calculates the index on this basis. Refer to the studies of relevant scholars [27–29], meanwhile, considering the availability of data and the actual situation of financial exclusion in China, this paper constructs an indicator system of inclusive finance from three dimensions: penetration of inclusive finance, utility of inclusive finance, and commercial sustainability of inclusive finance. Specific indicators are shown in Table 1.

**Table 1:** Inclusive finance indicator system.

Dimension	Indicator	Definition	Properties
**Penetration of inclusive finance**	Branch accessibility	Number of bank branches ÷Population	Positive
		Number of bank branches÷Geographic area	Positive
	Staff accessibility	Number of bank staff÷Population	Positive
		Number of bank staff÷Geographic Area	Positive
**Utility of inclusive finance**	Resident savings per capita	Savings ÷ Population	Positive
	Resident loans per capita	Loans ÷ Population	Positive
	Savings ratio	Savings ÷ GDP	Positive
	Loans ratio	Loans ÷ GDP	Positive
**Commercial sustainability of inclusive finance**	Non-performing loans ratio	Non-performing loans ÷ Loans	Negative
	Insurance loss ratio	Insurance indemnity expenditure ÷ Premium income	Negative

After the establishment of the inclusive finance indicator system, it is necessary to determine the weight of each indicator and even dimension, and then synthesize the inclusive finance index. This paper uses the coefficient of variation method to measure the inclusive finance index. Since the units of each indicator in the indicator system of inclusive finance are different, it is necessary to carry out dimensionless treatment for each indicator before calculating the index of inclusive finance. After the dimensionless treatment, the value range of each indicator is guaranteed to be [0,1]. The specific formula is as follows:

$$x_{ij}=\frac{A_{ij-m_{ij}}}{M_{ij-m_{ij}}}$$ (11)

$$x_{ij}=\frac{M_{ij-A_{ij}}}{M_{ij-m_{ij}}}$$ (12)

Where, *x*_*ij*_ represents the indicator value after processing, *A*_*ij*_ represents the indicator value before normalization processing, *m*_*ij*_ represents the minimum value of this indicator, and *M*_*ij*_ represents the maximum value of this indicator. When the indicator is positive, Eq (11) shall be used for measurement. However, when the indicator is negative, Eq (12) shall be used for measurement. Since every indicator in the indicator system of inclusive finance constructed in this paper is positive, Formula (11) only needs to be used to standardize each indicator.

Then, it calculates the inclusive finance index under the single dimension. Calculate the Euclidean distance between the measured value of each dimension and the optimal value, and integrate all distances together. The specific calculation formula is shown as follows:

$${IFI}_{i}=1-\frac{\sqrt{w_{i1}^{2}{\left(1-x_{i1}\right)}^{2}+w_{i2}^{2}{\left(1-x_{i2}\right)}^{2}+\cdots +w_{ij}^{2}{\left(1-x_{ij}\right)}^{2}}}{\sqrt{\left(w_{i1}^{2}+w_{i2}^{2}+\cdots w_{ij}^{2}\right)}}$$ (13)

Where, *i* represents the *i*th dimension, *j* represents the *j*th indicator under this dimension, and *w*_*ij*_ represents the weight of the *j*th indicator under the *i*th dimension. *w*_*ij*_ is calculated as $w_{ij}=\frac{V_{ij}}{\sum_{j}V_{ij}}$, and *V*_*ij*_ represents the coefficient of variation of the *j*th indicator in the *i*th dimension. The calculation formula of *V*_*ij*_ is $V_{ij}=\frac{S_{ij}}{A_{ij}}$, and *S*_*ij*_ represents the standard deviation of the *j*th indicator under the *i*th dimension, and *A*_*ij*_ represents the average value of the *j*th indicator under the *i*th dimension.

Finally, the inclusive finance index under the composite dimension is calculated by the following formula:

$$IFI=1-\frac{\sqrt{w_{1}^{2}{\left(1-{IFI}_{1}\right)}^{2}+w_{2}^{2}{\left(1-{IFI}_{2}\right)}^{2}+w_{3}^{2}{\left(1-{IFI}_{3}\right)}^{2}}}{\sqrt{w_{1}^{2}+w_{2}^{2}+w_{3}^{2}}}$$ (14)

Where, *w*_1_, *w*_2_ and *w*_3_ respectively represent the weight of inclusive finance index in each dimension, and *IF*_1_, *IF*_2_ and *IF*_3_ respectively represent the inclusive finance index in the dimension of penetration of inclusive finance, utility of inclusive finance, and commercial sustainability of inclusive finance.

#### Calculation of industrial structure upgrading

The UIS refers to the process in which production factors and resources are reconfigured among various economic departments in rural areas to gradually realize Pareto improvement, achieve coordinated development among rural industries and improve production efficiency. This paper uses the research of Gan et al. (2011) for reference to measure the UIS from two dimensions the Rationalization of Industrial Structure(RIS) and the Advanced Industrial Structure(AIS) [30].

The RIS measures the coupling degree between the input structure and the output structure of agricultural factors, which not only reflects the coordination degree among various rural industries, but also reflects the effective utilization degree of resources of the whole rural industry. In this paper, the method of measuring the RIS is combined with the structure deviation degree method and the Hamming approach degree method. The rationalization index of industrial structure calculated by this method is a positive index, which also takes into account the relative importance of various industries in rural areas. The specific calculation formula is as follows:

$$RIS=1-\frac{1}{4}\sum_{i=1}^{4}|S_{i}^{y}-S_{i}^{l}|$$ (15)

Where, RIS represents the rationalization index of industrial structure, $S_{i}^{y}=\frac{Y_{i}}{Y}、S_{i}^{l}=\frac{L_{i}}{L}$, Y_i_ represents the added value of industry i (i respectively refers to agriculture, forestry, animal husbandry and fishery), Y represents the added value of the primary industry. L_i_ represents the number of employed people in industry i, and L represents the total number of employed people in the primary industry. Then Y_i_/Y represents the output structure and is respectively represented by the proportion of the added value of agriculture, forestry, animal husbandry and fishery in the added value of the primary industry; L_i_/L represents the employment structure and is respectively represented by the proportion of the employed people of agriculture, forestry, animal husbandry and fishery in the primary industry. It should be noted that, since the statistical yearbook only published the total number of employed people in the primary industry, and did not publish the number of employment in agriculture, forestry, animal husbandry, fishing and other industries, this paper chose their median consumption value to replace the number of employment to measure the employment structure of agriculture. The rationalization index of industrial structure measured by this method is positive. The greater the value of this index is, the higher the degree of rationalization of industrial structure is. When the value of this index is 1, it indicates that the rural economy is in a state of balanced development.

The AIS refers to the process of the transformation of industrial structure from low level to high level. In this paper, the ratio between the output value of agricultural processing industry and that of agriculture, forestry, animal husbandry and fishery industry is used as the index to measure the AIS. This index can clearly reflect the degree of industrial technology penetration into agricultural industry. The higher the value of this index is, the higher the degree of processing of agricultural economy is, and the higher the degree of the AIS is. The industry scope of agricultural processing industry includes 12 types of industries. They are agricultural and sideline food processing industry, food manufacturing industry, wine and beverage and refined tea manufacturing industry, tobacco products, textiles, textile clothing and apparel, leather and fur industry, wood processing and bamboo, wood and rattan industry, furniture manufacturing industry, paper and paper products, printing and recording media reproduction industry, rubber and plastic products respectively. The specific calculation formula is as follows:

$$AIS=\frac{X_{2}}{X_{1}}$$ (16)

Where *AIS* represents the upgrading index of industrial structure, X_2_ and X_1_ represent the output value of agricultural processing industry and the output value of agriculture, forestry, animal husbandry and fishery respectively.

#### Control variables

For eliminating the influence of other factors on the results, refer to the study of Shimamoto et al.(2015) [31], Li et al. (2016) [32], Liu and Liu (2016) [33], and other scholar, the degree of government intervention, level of informatization, level of human capital, level of urbanization, level of fixed asset investment, population density and so on were included as control variables in the econometric model. These control variables are relevant factors that have important influence on farmers’ income. (1) Degree of government intervention. Public expenditure is an important factor affecting regional output [34]. The government can influence the improvement of farmers’ income by controlling the investment scale and direction of public financial resources. In this paper, the proportion of fiscal expenditure in GDP of each region is used as an indicator of the degree of government intervention. (2) Level of informatization. Information communication technologies can improve farmers’ lives by providing market information to create new opportunities of employment, education and learning for farmers [35]. In this paper, the informatization level of each region is measured by the total amount of postal and telecommunications services per capita. (3) Level of human capital. Human capital can improve agricultural production efficiency by affecting the allocation of agricultural production factors [36], and agricultural production efficiency is closely related to farmers’ income. This paper uses the ratio of teachers to students in colleges and universities to measure the level of human capital. (4) Level of urbanization. On the one hand, the improvement of urbanization level can promote the transfer of rural surplus labor to cities and promote the rational allocation of labor resources [37]. On the other hand, it also lays a foundation for agricultural scale management and contributes to the improvement of agricultural production efficiency. This paper uses the proportion of urban population to total population to measure the level of urbanization. (5) Level of fixed asset investment. Fixed asset investment can directly lead to economic growth through the multiplier effect [38], which plays an important role in promoting agricultural development and increasing farmers’ income. This paper uses per capita fixed asset investment to measure the level of fixed asset investment. (6) Population density. The increase of population density is conducive to the reduction of economic costs caused by infrastructure construction and public service supply [39], which is closely related to resources and economy. This paper uses the population per unit land area to measure the population density of each region.

#### Model setting

Based on the analysis of theoretical mechanism above, this paper first builds a fixed effect model of panel data to test the impact of inclusive finance on farmers’ income:

$${REV}_{it}=\alpha_{0}+\alpha_{1}{IFI}_{it}+\alpha_{2}{GOV}_{it}+\alpha_{3}{IFO}_{it}+\alpha_{4}{MPI}_{it}+\alpha_{5}{URB}_{it}+\alpha_{6}{IFA}_{it}+\alpha_{7}{POP}_{it}+u_{i}+\varepsilon_{it}$$ (17)

In Formula (17), *i* represents the specific province, *t* represents the specific year, *α*_0_ is a constant term, *REV* is the explained variable, represents the income level of farmers, and is represented by the per capita disposable income of rural residents. *IFI* is an explanatory variable, representing the development level of inclusive finance. It is measured by the synthetic index of inclusive finance, which is subdivided into three dimensions: penetration degree of inclusive finance, utility degree of inclusive finance, and business sustainability degree of inclusive finance. The control variables are as follows: *GOV* represents the degree of government intervention in economic development. *IFO* represents the level of informatization. *MPI* represents the level of human capital. *URB* represents the level of urbanization. *IFA* refers to the level of fixed asset investment. *POP* is the population density. *u*_*i*_ is an unobservable regional effect and *ε*_*it*_ is a random disturbance term. The specific definition of each variable is shown in Table 2.

**Table 2:** Variable definition.

Variable type	Variable name	Variable definition
**Explained variables**	REV	Gross farmer’s income ÷ Rural population
**Explanatory variables**	IFI	Synthetic index
	IFI_1_	Synthetic index
	IFI_2_	Synthetic index
	IFI_3_	Synthetic index
**Mediating variables**	RIS	Synthetic index
	AIS	Agro-industry output value÷Agriculture, forestry, animal husbandry and fishery output value
**Control variables**	GOV	Fiscal expenditure ÷ GDP
	IFO	Total postal and telecommunications business ÷ Population
	MPI	Number of college teachers ÷Number of undergraduates in colleges
	URB	Urban population ÷ Population
	IFA	Fixed asset investment ÷ Population
	POP	Population÷Geographic area

For the purpose of further verifying whether the UIS is the transmission mechanism of inclusive finance affecting farmers’ income, that is, whether inclusive finance promotes the UIS to increase farmers’ income. We references the testing procedures of mediation effect proposed by Baron and Kenny (1986) [40] set mediation effect model, based on the reference model (17) type, as follows:

$${UIS}_{it}=\beta_{0}+\beta_{1}{IFI}_{it}+\beta_{2}{GOV}_{it}+\beta_{3}{IFO}_{it}+\beta_{4}{MPI}_{it}+\beta_{5}{URB}_{it}+\beta_{6}{IFA}_{it}+\beta_{7}{POP}_{it}+u_{i}+\varepsilon_{it}$$ (18)

$${REV}_{it}=\pi_{0}+\pi_{1}{IFI}_{it}+\pi_{2}{UIS}_{it}+\pi_{3}{GOV}_{it}+\pi_{4}{IFO}_{it}+\pi_{5}{MPI}_{it}+\pi_{6}{URB}_{it}+\pi_{7}{IFA}_{it}+\pi_{8}{POP}_{it}+u_{i}+\varepsilon_{it}$$ (19)

In Eqs (18) and (19), *β*_0_ and *π*_0_ are constant terms, and the intermediate variable is the UIS, including two dimensions, namely the RIS and AIS, which are measured by the rationalization index and advanced index of industrial structure. According to the study of Baron and Kenny (1986) [40], the testing procedure of the intermediating effect is as follows: step one is to test the significance of the coefficient *α*_1_ in Eq (17). If it is significant, it is suspected to be an intermediating effect, and proceed to the second step. The second step is to observe whether the coefficients *β*_1_ and *π*_2_ are significant at the same time. If both are significant, it means that the influence of the development of inclusive finance on farmers’ income is at least partially realized through the UIS as an intermediary variable. The third step is to observe whether the influence coefficient *π*_1_ of inclusive finance on farmers’ income is significant. If it is significant and has the same sign with *β*_1_*π_2_, it proves that there is a partial intermediating effect, which means that only part of the influence of inclusive finance on farmers’ income is realized through the UIS as the mediating variable. If *π*_1_ is not significant, it indicates that there is a complete intermediating effect, that is, inclusive finance affects farmers’ income completely through UIS. The meaning of the other variables is described above.

#### Descriptive statistics

Before the following empirical analysis, we first conducted descriptive statistics on each variable, and the descriptive statistics results of specific sample are shown in Table 3. During the sample period from 2006 to 2016, the average value of explained variable, that is REV, was 0.817, the minimum value was 0.198 but the maximum value was as high as 2.552, indicating that there were great differences in income levels among different farmers. The average value of the explanatory variable, IFI, is 0.118. Specifically, from the three dimensions of inclusive finance, the average value of IFI_3_ is 0.660, which is about 6 times than that of IFI_1_ and IFI_2_. The average values of IFI1 and IFI_2_ are 0.100 and 0.147, respectively. The average values of RIS and AIS were 0.929 and 2.226, respectively. The average values of GOV indicates that fiscal spending as a share of GDP was about 20% during the sample period. The average values of URB reflects an average urbanization rate of 0.537 in China during the sample period, indicating that about half of the total population is urban. In addition, the average values of IFO, MPI, IFA and POP are 0.171, 0.101, 2.617 and 0.468, respectively.

**Table 3:** Sample descriptive statistics.

Variable	Mean	St. D	Min	Max	Obs
**REV**	0.817	0.434	0.198	2.552	308
**IFI**	0.118	0.127	0.026	0.801	308
**IFI**_**1**_	0.100	0.143	0.010	0.952	308
**IFI**_**2**_	0.147	0.146	0.010	0.975	308
**IFI**_**3**_	0.660	0.139	0	0.982	308
**RIS**	0.929	0.035	0.847	0.996	308
**AIS**	2.226	2.457	0.248	15.151	308
**GOV**	0.205	0.073	0.083	0.429	308
**IFO**	0.171	0.103	0.058	0.626	308
**MPI**	0.101	0.029	0.070	0.239	308
**URB**	0.537	0.139	0.275	0.896	308
**IFA**	2.617	1.512	0.319	8.181	308
**POP**	0.468	0.649	0.012	3.826	308

#### Analysis results of farmers’ income

Table 4 reports the average income of farmers in 28 provinces of China from 2006 to 2016. In the sample period, the average income of farmers in the eastern, central and western regions is 1.081, 0.711 and 0.589 respectively, showing a decreasing trend from east to west, and farmers’ income in the eastern region is significantly higher than that in the central and western regions. From the provincial level, the highest average value of farmers’ income in Shanghai is 1.641, while the lowest average value of farmers’ income in Guizhou is only 0.456, the highest value is 3.6 times of the lowest value, which shows that there are great differences in the farmers’ income between different provinces in China. Compared with the central and western regions, the difference of farmers’ income between different provinces in the eastern region is greater. Although Hebei and Hainan are located in the eastern region, the average income of farmers is only 0.741 and 0.693, which is significantly lower than that of other eastern regions, and also lower than that of Jilin, Heilongjiang and Hubei in the central regions.

**Table 4:** Average value of farmers’ income of 28 provinces.

Region	Rev	Region	Rev	Region	Rev
Beijing	1.496	Shanxi	0.613	Inner Mongolia	0.706
Tianjin	1.252	Jilin	0.766	Guangxi	0.589
Hebei	0.741	Heilongjiang	0.756	Chongqing	0.673
Liaoning	0.833	Anhui	0.677	Sichuan	0.655
Shanghai	1.641	Jiangxi	0.727	Guizhou	0.456
Jiangsu	1.112	Henan	0.700	Yunnan	0.512
Zhejiang	1.393	Hubei	0.745	Shaanxi	0.535
Fujian	0.927	Hunan	0.706	Ningxia	0.584
Shandong	0.866	Central average	0.711	Xinjiang	0.595
Guangdong	0.942			Western average	0.589
Hainan	0.693				
Eastern average	1.081				

**Fig 1:**
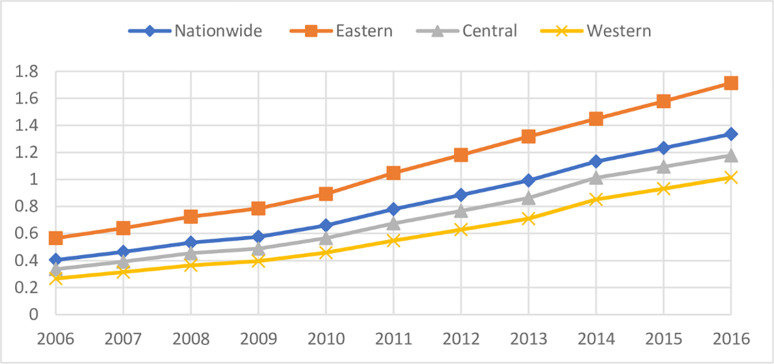
Time trend of farmers’ income of 28 provinces.

Fig 1 depicts the trend of the average income of farmers from 2006 to 2016.No matter from the perspective of the whole country or from the eastern, central and western regions, the average income of farmers showed a continuous upward trend from 2006 to 2016, which shows that the income situation of farmers in the eastern, central and western regions of China has been effectively improved. However, there are great differences in the farmers’ income in different regions. It can be seen from the time trend chart that farmers’ income in the eastern region is significantly higher than that in the central and western regions, and the gap is gradually widening; The income gap between the central region and the western region is not large, and the increase rate is relatively close in the sample period.

### Empirical results and analysis

#### Unit root test and cointegration test

In order to avoid the phenomenon of "pseudo regression", we first carried out the unit root test and cointegration test on the data before regression analysis. To ensure the validity of the unit root test, we use IPS test, HT test and Breitung test at the same time to test whether there is a unit root. The results show that there are unit roots in the horizontal values of all selected variables (Table 5).We further performed the unit root test on the first-order difference of variables, and the results showed that there was no unit root in each variable at the significance level of 1% or 5% (Table 6).It can be seen that the sequence has stationarity. Cointegration test can be used to test whether there is a cointegration relationship between variables. We also use Pedroni test and Westerlund test to test whether there is a long-term stable relationship between dependent variables and independent variables (Table 7).The results show that the two test methods reject the original hypothesis at the significance level of 1% and 10% respectively, indicating that there is a cointegration relationship between the variables, which can be used for regression analysis of panel data.

**Table 5:** Unit root test.

Variable	Test		
	IPS	HT	Breitung
**Rev**	-0.131	1.017	8.344
**IFI**	-1.109	0.914	7.759
**RIS**	-2.938*	0.423***	0.024
**AIS**	-0.661	0.932	3.577
**GOV**	-1.610	0.689	0.606
**IFO**	-1.339	0.564***	-2.882***
**MPI**	-1.483	0.800	3.367
**URB**	-0.487	0.943	6.250
**IFA**	-0.173	0.961	4.694
**POP**	-1.274	0.836	5.520

Note

* p<0.1, ** p<0.05

*** p<0.01.

**Table 6:** First-order difference unit root test.

Variable	Test		
	IPS	HT	Breitung
**Rev**	-2.491***	0.017***	-7.574***
**IFI**	-3.253***	-0.467***	-6.743***
**RIS**	-4.774***	-0.211***	-3.252***
**AIS**	-2.956***	-0.203***	-8.961***
**GOV**	-2.588***	0.003***	-6.402***
**IFO**	-2.782***	0.024***	-7.107***
**MPI**	-2.695***	0.060***	-4.362***
**URB**	-2.530***	-0.026***	-5.838***
**IFA**	-1.790**	0.443***	-3.072***
**POP**	-2.447***	-0.020***	-8.925***

Note:* p<0.1

** p<0.05

*** p<0.01.

**Table 7:** Cointegration test.

Test	Statistic name	Statistic value
**Pedroni**	Modified Phillips-Perron t	9.581***
	Phillips-Perron t	-20.471***
	Augmented Dickey-Fuller t	-17.494***
**Westerlund**	Variance ratio	1.418*

Note

* p<0.1, ** p<0.05

*** p<0.01.

#### Regression results and analysis of benchmark model

This paper first tests the impact of inclusive finance on farmers’ income. Column (1) and (2) of Table 8 respectively report the regression results of the impact of inclusive finance on farmers’ income without and after the addition of control variables. As can be seen from the regression results, inclusive finance can significantly promote the increase of farmers’ income, and it is significant at the level of 1%. This shows that the higher the level of inclusive finance development, the more beneficial it is to increase the income of local farmers. Specifically, as shown in Column (2), the coefficient of inclusive finance (IFI) is 6.733, indicating that the income level of farmers increases by 6.733 units for every 1 unit increase in the level of inclusive finance development.

**Table 8:** Benchmark regression results.

Variable	REV							
	(1)	(2)	(3)	(4)	(5)	(6)	(7)	(8)
**IFI**	8.145***(0.323)	6.733***(0.389)						
**IFI**_**1**_			6.026***(0.521)	2.623***(0.486)				
**IFI**_**2**_					4.685***(0.128)	2.566***(0.161)		
**IFI**_**3**_							-0.969***(0.140)	-0.116**(0.053)
**GOV**		0.450*(0.247)		1.802***(0.322)		-0.162 (0.273)		1.962***(0.341)
**IFO**		-0.337***(0.083)		-0.057(0.111)		-0.253***(0.085)		0.054 (0.116)
**MPI**		4.630***(1.015)		8.360***(1.361)		1.179(1.132)		8.361*** (1.429)
**URB**		1.155***(0.270)		1.808***(0.369)		1.342***(0.280)		2.041*** (0.383)
**IFA**		0.106***(0.008)		0.146***(0.011)		0.095***(0.009)		0.140*** (0.012)
**POP**		-0.742***(0.124)		0.359**(0.173)		0.326***(0.078)		1.180*** (0.076)
**Cons**	-0.145***(0.040)	-1.029***(0.208)	0.214***(0.054)	-2.168***(0.268)	0.128***(0.020)	-0.724***(0.227)	1.457***(0.094)	-2.375***(0.281)
**Obs**	308	308	308	308	308	308	308	308
**R** ^**2**^	0.695	0.954	0.324	0.913	0.827	0.950	0.147	0.905

Note

***, **, and * indicate the significance levels of 1%, 5% and 10%, respectively. Robust standard errors are shown in parentheses, and the following table is the same.

Considering that different dimensions of inclusive finance may have different influences on farmers’ income, this paper further investigates the impact of inclusive finance on farmers’ income from three dimensions, namely, the penetration dimension of inclusive finance, the utility dimension of inclusive finance, and the commercial sustainability dimension of inclusive finance. The regression results are shown in columns (3) to (8) of Table 8, where columns (3), (5) and (7) are regression results without the addition of control variables, but columns (4), (6) and (8) are regression results after the addition of control variables. It can be found from the regression results that both the penetration dimension of inclusive finance (IFI_1_) and the utility dimension of inclusive finance (IFI_2_) can significantly promote the increase of farmers’ income and pass the significance level test of 1%, while the commercial sustainability dimension of inclusive finance(IFI_3_) does not significantly promote the increase of farmers’ income. These regression results show that with the improvement of inclusive finance penetration dimension and inclusive finance utility dimension, farmers’ income level gradually increases, while inclusive finance commercial sustainability dimension does not show positive effects on farmers’ income increase. The penetration of inclusive finance reflects the breadth of service coverage of financial institutions. Improving the penetration of inclusive finance is the most basic requirement for the development of inclusive finance, which helps to reduce the contact exclusion of small and micro enterprises, farmers and other vulnerable groups to financial services. The utility of inclusive finance reflects the depth of the use of inclusive finance services by the demand side, which directly reflects the access and use of financial services by small and micro enterprises, farmers and other vulnerable groups. Therefore, the penetration and utility dimension of inclusive finance can significantly promote the increase of farmers’ income. The business sustainability dimension of inclusive finance mainly describes the sustainability of financial institutions’ popularization of financial services. This dimension is to ensure that financial institutions should not completely sacrifice their own interests to improve the level of financial inclusiveness. The sustainable development of inclusive finance requires a good external environment [41]. The commercial sustainability of inclusive finance is a long-term concern of financial institutions and regulators, so it may not be an important factor to promote the increase of farmers’ income in the short term.

#### Regression results and analysis of intermediating effect model

In order to deeply analyzing the relationship among inclusive finance, industrial structure upgrading and farmers’ income, this paper empirically tested whether industrial structure upgrading is the intermediary mechanism of inclusive finance affecting farmers’ income based on the intermediary effect test procedure proposed by Baron and Kenny (1986) [40]. Table 9 shows the regression results of the intermediating effect of inclusive finance on farmers’ income. Since it has been found in the benchmark regression results that inclusive finance can significantly promote the increase of farmers’ income, this part directly enters the second and third steps of the intermediation effect test procedure.

**Table 9:** Inclusive finance and farmers’ income: Mechanism test.

Variable	RIS (1)	Rev (2)	AIS(3)	Rev(4)
**IFI**	0.104** (0.041)	6.567*** (0.389)	11.670*** (3.016)	6.471*** (0.394)
**RIS**		1.594*** (0.573)		
**AIS**				0.022***(0.008)
**Controlling Variables**	YES	YES	YES	YES
**Obs**	308	308	308	308
**R** ^**2**^	0.136	0.955	0.738	0.955

Note:* p<0.1

** p<0.05

*** p<0.01.

Column (1) and (2) of Table 9 are the regression results of intermediating effect with RIS as the mediating variable. Column (1) is listed as the regression result of the influence of inclusive finance on the RIS. It can be seen from the regression result that the estimated coefficient of inclusive finance is significantly positive and has passed the significance level test of 5%, indicating that the development of inclusive finance can significantly promote the improvement of the RIS. Column (2) is listed as the regression result of inclusive finance affecting farmers’ income through the mediating variable, the RIS. In this result, the estimated coefficient of inclusive finance is significantly positive at the 1% level, and the estimated coefficient of mediating variable, RIS, is significantly positive at 1% level. It is indicating that the RIS has a partial intermediating effect between inclusive finance and farmers’ income. Column (3) and (4) are the regression results of intermediating effect with the AIS as the mediating variable. Column (3) shows the regression results of the inclusive finance affecting the AIS. It can be seen from Column (3) that the estimated coefficient of inclusive finance is significantly positive at the 1% level, indicating that the development of inclusive finance can bring about the improvement of the AIS. Column (4) is listed the regression result of inclusive finance affecting farmers’ income through the intermediary variable, AIS. In Column (4), the estimated coefficient of inclusive finance is significantly positive at the 1% level, and the estimated coefficient of intermediary variable, the AIS, is significantly positive at the 1% level, indicating that the AIS has a partial intermediating effect between inclusive finance and farmers’ income.

The above regression results show that the UIS is the intermediating mechanism for inclusive finance to increase farmers’ income. Moreover, there is a transmission mechanism that the development of inclusive finance promotes the UIS, and then the UIS promotes the increase of farmers’ income. The possible reason is that inclusive finance can improve farmers’ ability to use credit funds for production and operation and market participation by exerting the “hematopoietic function” and “guiding role” of finance, and guide financial resources to the industries and departments with higher economic efficiency. As a result, inclusive finance realizes the purpose to promote the economic transformation and adjustment of rural areas and the UIS. Further, the UIS can promote the improvement of rural industrial production efficiency and stimulate the vitality of rural industrial development, thereby increasing the income opportunities and broadening the channels of income sources of farmers.

To further analyze whether the UIS is still the intermediary mechanism for inclusive finance to promote the increase of farmers’ income, if farmers are at different income levels, this paper further conducts quantile regression of panel data. Five quantiles, 10%, 25%, 50%, 75% and 90%, were selected to correspond to the lowest-income group, middle-and-low-income group, middle-income group, middle-and-high-income group and highest-income group of rural residents. Table 10 illustrates the regression results of the intermediating effect of inclusive finance on income of farmers, under different income levels.

**Table 10:** Regression results of mechanism heterogeneity.

Coefficients		10%	25%	50%	75%	90%
***α***_**1**_	**RIS**	6.060*** (0.833)	6.338*** (0.615)	6.662*** (0.518)	7.202*** (0.823)	7.541*** (1.146)
	**AIS**	6.060*** (0.833)	6.338*** (0.615)	6.662*** (0.518)	7.202*** (0.823)	7.541*** (1.146)
***π***_**1**_	**RIS**	5.859*** (0.799)	6.149*** (0.591)	6.497*** (0.492)	7.051*** (0.760)	7.392*** (1.048)
	**AIS**	5.731*** (0.917)	6.012*** (0.694)	6.400*** (0.572)	6.981***(0.900)	7.332***(1.233)
***π***_**2**_	**RIS**	1.437* (0.817)	1.501** (0.605)	1.579*** (0.498)	1.702** (0.777)	1.778* (1.075)
	**AIS**	0.034*** (0.013)	0.030*** (0.010)	0.024*** (0.008)	0.015 (0.013)	0.009 (0.018)
**Controlling Variables**		YES	YES	YES	YES	YES
**Obs**		308				

Note: The influence coefficients of *α*_1_, *π*_1_ and *π*_2_ in the table are respectively used in the regression model of intermediating effect when the mediating variables are the RIS and AIS.

*α*_1_ is the regression coefficient of inclusive finance affecting farmers’ income. At different quantile levels, inclusive finance has a significant positive impact on farmers’ income and has passed the significance level test of 1%, which indicates that inclusive finance can significantly promote the income growth of farmers with different income levels. At the same time, it can be found that with the increase of quantile level, the influence coefficient of inclusive finance on farmers’ income gradually increases. It may be that compared to lowest income groups, farmers in the highest income group will further pursue factor accumulation and meet developmental needs rather than using credit funds only to meet survival needs, thereby creating more opportunities for themselves. Therefore, the promotion effect of inclusive finance on the income of farmers in the highest-income group is greater than that in the lowest-income group.

*β*_1_ is the regression coefficient of inclusive finance improving the RIS and the AIS. It has been proved that inclusive finance has a significant positive influence on both the RIS and the AIS. *π*_1_ and *π*_2_ are the regression coefficients of inclusive finance and mediating variables affecting farmers’ income when the mediating variables are respectively the RIS and the AIS. The influence coefficient *π*_1_ of inclusive finance on farmers’ income at different quantiles is significant, and it has passed the significance level test of 1%. The influence coefficient *π*_2_ of the RIS on farmers’ income of different quantiles is significant at 10%,25%,50%,75% and 90% quantiles, and *π*_1_ is with the same sign as *β*_1_*π_2_. The influence coefficient *π*_2_ of the AIS on farmers’ income is significant at 10%, 25% and 50% quantiles, and *π*_1_ is with the same sign as *β*_1_*π_2_. This indicates that the RIS has a partial intermediating effect on the relationship between inclusive finance and farmers’ income among all income groups. In the middle-income groups and groups with lower income, the AIS has a partial intermediating effect on the relationship between inclusive finance and farmers’ income. This demonstrates that the UIS, as an intermediary mechanism for inclusive finance to promote farmers’ income increase, has heterogeneity among farmers with different income levels.

#### Endogeneity problem and robustness test

*Endogeneity problem*. This paper finds that the UIS is the intermediary mechanism of inclusive finance affecting farmers’ income. Endogeneity problems can be caused by sample selection bias, mutual causality of variables and omission of variables. To minimize the estimation errors caused by endogeneity problems and ensure the relative reliability of the research conclusions, this paper carried out a period lag for all explanatory variables, mediating variables and control variables, and re-used the fixed-effect model of panel data for regression test. Table 11 illustrates the fixed effect regression results with a period lag. The regression results show that inclusive finance can increase farmers’ income, and the coefficient passes the significance level test of 1%. Also, inclusive finance has a significant positive impact on the RIS and AIS, and the coefficients have passed the significance level test of 1% and 10% respectively. Moreover, the RIS and AIS have significant positive effects on farmers’ income, respectively, and the coefficients have passed the significance level test of 1%, respectively. The regression results indicate that the RIS and AIS have a partial intermediating effect on the impact of inclusive finance on farmers’ income, and the UIS is the intermediary mechanism of inclusive finance driving farmers’ income increase. The regression results are completely consistent with the conclusions drawn from the original regression results, indicating that the research conclusions drawn above are robust.

**Table 11:** Endogenous test.

Variable	REV	RIS	REV	REV	AIS	REV
**L.IFI**	6.193*** (0.423)	0.135*** (0.042)	6.101*** (0.419)	6.193*** (0.423)	6.281* (3.388)	5.852*** (0.434)
**L.RIS**			1.538*** (0.560)			
**L.AIS**						0.022*** (0.008)
**Controlling Variables**	*YES*	*YES*	*YES*	*YES*	*YES*	*YES*
**Obs**	280	280	280	280	280	280
**R** ^**2**^	0.960	0.178	0.961	0.960	0.766	0.961

Note

:* p<0.1, ** p<0.05

*** p<0.01.

*Robustness test*: *Add control variables*. In benchmark regression model, this paper only controls the degree of government intervention (GOV), informationization level (IFO), human capital level (MPI), the urbanization level (URB), the level of investment in fixed assets (IFA), the density of population (POP) and other variables. In order to test the robustness of the regression results, other variables that may affect farmers’ income, such as regional openness level (OPE), education level (EDU) and infrastructure development level (FRA), are added to the original control variables, and the model is re-estimated. The OPE is measured by the proportion of the total import and export trade of each province to GDP, the EDU represented by the proportion of the total number of college students in each province to the number of total population, and the FRA is measured by the per capita highway mileage. Table 12 shows the regression results after adding control variables. As can be seen from the regression results, after the addition of other control variables, inclusive finance can still increase farmers’ income, and the coefficient has passed the significance level test of 1%. Inclusive finance has a significant positive impact on the RIS and AIS, and the coefficients have passed the significance level test of 5% and 1%, respectively. Both the RIS and AIS have significant positive effects on farmers’ income, and the coefficients have passed the significance level test of 1% and 5%. The regression results indicate that the RIS and AIS still have a partial intermediating effect in the process of inclusive finance affecting farmers’ income, that is, the UIS is the intermediary mechanism of inclusive finance increasing farmers’ income. The regression results are completely consistent with the original regression results, which once again proves the robustness of the research conclusions drawn above.

**Table 12:** Robustness test: Add control variables.

Variable	Rev	RIS	Rev	Rev	AIS	Rev
**IFI**	6.912*** (0.433)	0.096** (0.051)	6.752*** (0.430)	6.912*** (0.433)	9.519*** (3.326)	6.744*** (0.436)
**RIS**			1.656*** (0.561)			
**AIS**						0.018** (0.008)
**GOV**	0.502** (0.246)	0.003 (0.026)	0.497** (0.242)	0.502** (0.246)	-4.025** (1.886)	0.573** (0.246)
**IFO**	-0.320*** (0.082)	-0.004 (0.009)	-0.313*** (0.081)	-0.320*** (0.082)	-2.103*** (0.632)	-0.283*** (0.083)
**MPI**	2.803* (1.455)	-0.067 (0.156)	2.914** (1.436)	2.803* (1.455)	-10.733 (11.177)	2.992** (1.447)
**URB**	1.517*** (0.321)	0.029 (0.034)	1.470*** (0.317)	1.517*** (0.321)	-4.451* (2.469)	1.596*** (0.321)
**IFA**	0.106*** (0.009)	-0.001(0.001)	0.107***(0.009)	0.106***(0.009)	0.444***(0.067)	0.098***(0.009)
**POP**	-0.838***(0.139)	-0.015(0.015)	-0.813***(0.138)	-0.838***(0.139)	4.524***(1.070)	-0.917***(0.143)
**OPE**	0.033(0.066)	-0.003 (0.007)	0.038 (0.065)	0.033 (0.066)	-0.703 (0.505)	0.046 (0.065)
**EDU**	-3.641 (5.775)	-0.154 (0.618)	-3.386 (5.695)	-3.641 (5.775)	59.403 (44.350)	-4.686 (5.752)
**FRA**	-0.008*** (0.002)	0.0001 (0.0002)	-0.008*** (0.002)	-0.008*** (0.002)	-0.062*** (0.017)	-0.007*** (0.002)
**Cons**	-0.726*** (0.259)	0.918*** (0.028)	-2.246*** (0.575)	-0.726*** (0.259)	3.585* (1.988)	-0.789*** (0.259)
**Obs**	308	308	308	308	308	308
**R** ^**2**^	0.956	0.137	0.958	0.956	0.756	0.957

Note: OPE, EDU and FRA represent the level of regional openness, education development and infrastructure development respectively.

*Robustness test*: *Change the measurement method of UIS and sample time interval*. In the original test, we use the structure deviation method and Hamming closeness method to measure RIS, and use the ratio of the output value of agricultural products processing industry to the output value of agriculture, forestry, animal husbandry and fishery to measure AIS. The sample period of the study is from 2006 to 2016. In order to enhance the credibility of the results of this study, we remeasure the UIS, and set the sample period of the study from 2006 to 2018 to perform the robustness test again.

Firstly, we refer to the research of Gan et al. (2011) [30] and use the Theil index method to measure RIS. The specific calculation formula is as follows:

$$RIS=\sum_{i=1}^{n}\frac{Y_{i}}{Y}\ln\left(\frac{Y_{i}}{L_{i}}/\frac{Y}{L}\right)$$ (20)

The meaning of each indicator in the formula is consistent with that in the original measurement method. It should be noted that the index of the RIS measured by Theil index is reverse. The lower the index value is, the higher the rationalization degree of industrial structure is, and it indicates that the agricultural economy is in a state of balanced development when the index value is 0.

Then, we use the idea of Zhou (2017) [42] to measure AIS by using the proportion of the output value of agriculture, forestry, animal husbandry and fishery service industry in the output value of agriculture, forestry, animal husbandry and fishery.

Table 13 shows the regression results after changing the measurement method of UIS and sample time interval. It can be seen from the regression results that inclusive finance has a significant positive impact on farmers’ income, and the coefficient has passed the significance level test of 1%. Inclusive finance has a significant negative and positive impact on the RIS and AIS respectively, and the coefficients have passed the significance level test of 5% and 1%, respectively. Since the re-measured index of the RIS is reverse, the regression results show that inclusive finance can significantly promote the RIS and AIS, that is, inclusive finance can help promote the UIS. Both the RIS and AIS can significantly increase farmers’ income, and the coefficients have passed the significance level test of 1% respectively. This shows that the UIS is the intermediary mechanism of inclusive finance to promote the increase of farmers’ income. The regression results are completely consistent with the original regression results, which once again proves the robustness of the research conclusions.

**Table 13:** Robustness test: Change the measurement method of UIS and sample time interval.

Variable	REV	RIS	REV	REV	AIS	REV
**IFI**	7.701*** (0.436)	-0.117** (0.059)	7.502*** (0.427)	7.701*** (0.436)	0.103*** (0.030)	7.375*** (0.434)
**RIS**			-1.703*** (0.397)			
**AIS**						3.149*** (0.795)
**Controlling Variables**	*YES*	*YES*	*YES*	*YES*	*YES*	*YES*
**Obs**	364	364	364	364	364	364
**R** ^**2**^	0.949	0.184	0.951	0.949	0.268	0.951

Note:* p<0.1

** p<0.05

*** p<0.01.

### Further study

This paper builds a threshold model of panel fixed effect by reference to Wang (2015) [43], and studies the threshold effect of the UIS affecting farmers’ income under the influence of inclusive finance. It aims to further explore whether there are differences in the promotion intensity of the UIS on farmers’ income under different development levels of inclusive finance. Based on the intermediating effect model above, this paper introduces the indicator function, and takes IFI as the threshold variable to construct the single-threshold model (21) and multi-threshold model (22) of the panel data respectively. The panel threshold models are set as follows:

$${Rev}_{it}=\alpha_{0}+\alpha_{1}{IFI}_{it}+\alpha_{2}{UIS}_{it}*I({IFI}_{it}<\gamma_{1})+\alpha_{3}{UIS}_{it}*I({IFI}_{it}\geq \gamma_{1})+\alpha_{4}{GOV}_{it}+\alpha_{5}{IFO}_{it}+\alpha_{6}{MPI}_{it}+\alpha_{7}{URB}_{it}+\alpha_{8}{IFA}_{it}+\alpha_{9}{POP}_{it}+u_{i}+\varepsilon_{it}$$ (21)

$${\mathrm{Rev}}_{it}=\alpha_{0}+\alpha_{1}IFI_{it}+\alpha_{2}UIS_{it}*I({\mathrm{IFI}}_{it}<\gamma_{1})+\alpha_{3}UIS_{it}*I(\gamma_{1}\leq IFI_{it}<\gamma_{2})+\ldots \ldots \alpha_{n+1}UIS_{it}*I(\gamma_{n-1}\leq IFI_{it}<\gamma_{n})+\alpha_{n+2}UIS_{it}*I(IFI_{it}\geq \gamma_{n})+\alpha_{n+3}GOV_{it}+\alpha_{n+4}IFO_{it}+\alpha_{n+5}MPI_{it}+\alpha_{n+6}URB_{it}+\alpha_{n+7}IFA_{it}+\alpha_{n+8}POP_{it}+u_{i}+\varepsilon_{it}$$ (22)

Before conducting empirical test, the number of thresholds needs to be determined in order to determine the specific form of threshold model. In this paper, a single threshold, a double threshold and a triple threshold were set for estimation respectively to obtain the F statistic and the P value under Bootstrap. Table 14 shows the corresponding threshold value estimation results when the mediating variables are respectively the RIS and AIS. The impact of the RIS on farmers’ income is affected by the double threshold value of inclusive finance, whose upper and lower limits are 0.137 and 0.195 respectively, and which pass the significance level test of 5%. The impact of the AIS on farmers’ income is affected by the single threshold value of inclusive finance, which is 0.137 and has passed the significance level test of 10%. Therefore, the Double panel threshold model and the single panel threshold model are respectively used to analyze the RIS and AIS.

**Table 14:** Threshold value estimation results.

Variable Type	Threshold Type	Threshold Value	F-Value	P-Value	Critical Value		
					10%	5%	1%
**RIS**	Single threshold	0.137	79.030***	0.000	27.711	32.159	42.069
	Double threshold	0.137/0.195	36.320**	0.030	28.118	33.123	42.337
	Triple threshold	0.528	33.970	0.170	52.589	65.567	91.326
**AIS**	Single threshold	0.137	43.640*	0.053	33.318	43.698	66.898
	Double threshold	0.137/0.394	26.710	0.130	29.253	33.639	54.136
	Triple threshold	0.223	18.120	0.287	30.485	40.296	58.676

Note:(1) Both P values and critical values were obtained by repeated sampling with Bootstrap method for 300 times.

(2) ***, ** and * represent significance at the level of 1%, 5% and 10%, respectively.

Table 15 shows the regression results for the panel thresholds. The regression results show that the RIS has a significant positive impact on farmers’ income, and this effect is affected by the double threshold of inclusive finance. When the inclusive finance is less than 0.137, the impact coefficient of the RIS on farmers’ income is 2.220, and it has passed the significance level test of 1%. When the inclusive finance is 0.137 and 0.195, the impact coefficient is 2.458, and it has passed the significance level test of 1%. And when the inclusive finance is greater than 0.195, the impact coefficient is 2.818, and it has passed the significance level test of 1%. The AIS has a significant positive impact on farmers’ income, and this effect is affected by the single threshold of inclusive finance. When the inclusive finance is less than 0.137, the impact coefficient of the AIS affecting farmers’ income is 0.004, it does not pass the significance test. However, when the inclusive finance is greater than 0.137, the impact coefficient is 0.057, and it has passed the significance level test of 1%. The above results indicate that, compared with the development level of low inclusive finance, the RIS and AIS have a stronger effect on increasing farmers’ income under the development level of high inclusive finance. In other words, the higher the development level of inclusive finance, the greater the promoting effect of the UIS on farmers’ income increase. The possible reason is that the utilization rate of investment in the UIS is different in the area under low and high development level of inclusive finance. More specifically, in areas with a low level of inclusive finance development, due to higher financial exclusion from rural industries, the adoption rate and effect of new technologies and new methods brought about by the UIS will be reduced, and the radiation effect of the UIS will be weakened. This makes the utilization efficiency of investment funds for industrial structure upgrades lower. Considering that the upgrading of the industrial structure has a significant role in promoting farmers’ continuous income growth, the reduced utilization efficiency of investment funds for the UIS will hinder the continued growth of farmers’ income. In areas with a relatively high level of inclusive finance development, the use of funds invested in the UIS will be guaranteed, which will further promote the growth of farmers’ income.

**Table 15:** Regression results for the panel thresholds.

Variable	Rev	
**RIS(IFI<0.137)**	2.220*** (0.672)	
**RIS(0.137≤IFI<0.195)**	2.458*** (0.667)	
**RIS(IFI ≥0.195)**	2.818*** (0.667)	
**AIS(IFI<0.137)**		0.004 (0.013)
**AIS(IFI ≥0.137)**		0.057*** (0.012)
**GOV**	1.629*** (0.272)	1.679*** (0.303)
**IFO**	-0.051 (0.093)	0.037 (0.102)
**MPI**	5.809*** (1.181)	7.392*** (1.299)
**URB**	2.010*** (0.321)	2.173*** (0.357)
**IFA**	0.119*** (0.010)	0.133***(0.012)
**POP**	1.839*** (0.144)	0.409***(0.132)
**Cons**	-4.470*** (0.654)	-2.070*** (0.264)
**Obs**	308	308
**R** ^**2**^	0.940	0.928

Note:* p<0.1, ** p<0.05

*** p<0.01.

## Conclusion and implication

In this paper, panel data is from 28 Chinese provinces from 2006 to 2016 as samples for our empirical analysis. We construct an index system of inclusive finance from three dimensions, namely, the penetration dimension, the utility dimension and the commercial sustainability dimension of inclusive finance respectively, and use the coefficient of variation method to measure the development level of inclusive finance in all provinces of China. We also measure the level of the UIS in China’s provinces from two aspects: the RIS and AIS. Based on the fixed effect model, intermediating effect model and threshold effect model of panel data, we conduct empirical analysis and study on the relationship among inclusive finance, the UIS and farmers’ income. Through empirical results, we found that, firstly, the development of inclusive finance can significantly promote the increase of farmers’ income. From the perspective of various dimensions of inclusive finance, the penetration dimension and the utility dimension of inclusive finance can significantly promote the increase of farmers’ income, while the commercial sustainability dimension does not show a positive effect on farmers’ income. Secondly, the UIS is the intermediary mechanism for inclusive finance to promote the increase of farmers’ income, and this intermediary mechanism shows heterogeneity among farmers with different income levels. Thirdly, we further discuss that the promotion effect of the UIS on farmers’ income is affected by the threshold effect of inclusive finance. Compared with the development level of low inclusive finance, the promotion of UIS on farmers’ income is stronger under the development level of high inclusive finance. In addition, we tested the endogenous and robustness of the study results by using a period lag of the study variables, adding other control variables and change the measurement method of UIS and sample time interval, and the results found that the robustness test was completely consistent with the research conclusions obtained from the original test.

According to the empirical research conclusions of this paper, the following policy recommendations are proposed: First, local governments and financial institutions should further promote the development level of inclusive finance, especially pay attention to the availability and use cost of financial products and services for farmers. At the same time, the credit mechanism should be innovated to create a sound financial ecological environment. Second, great attention should be paid to the important role of industrial structure upgrading in the relationship between inclusive finance and farmers’ income. Financial institutions should design differentiated financial products and services according to the different factor endowments and industrial characteristics of rural areas. In addition, they should plan the release of credit resources from a strategic perspective, focus on supporting the industries that are in line with regional comparative advantages and have good market development prospects, and improve the allocation efficiency of financial resources. Through the upgrading of the industrial structure in rural areas, the effect of inclusive finance on increasing farmers’ income will play a maximum role. Thirdly, inclusive finance should promote the vulnerable groups to fully understand the basic financial rights they enjoy and enhance their ability to use financial rights to increase their income. Therefore, financial education for farmers should be strengthened [44, 45]and promote the organic connection between farmers and modern agricultural industry, so that farmers can fully share the dividends brought by industrial structure upgrading. Finally, financial institutions should strengthen in-depth cooperation with relevant agro-related enterprises and government departments to jointly promote the construction and improvement of infrastructure in relevant industries in rural areas and surrounding areas, so as to provide a good external environment for better playing inclusive finance to support the upgrading of industrial structure in rural areas.

The research of this paper can provide inspiration and reference for policy formulation of inclusive finance and sustainable development of rural economy. Compared with other studies on the economic effects of inclusive finance, the contributions of our study are mainly reflected in the following aspects. First, we demonstrate the mechanism of inclusive finance promoting the increase of farmers’ income from the perspective of the UIS, providing a new perspective for the existing research on the mechanism of inclusive finance promoting farmers’ income. We use the intermediating effect model to confirm that the UIS is the intermediary mechanism for inclusive finance to promote farmers’ income increase, and further analyze the heterogeneity of this intermediary mechanism among farmers with different income levels. Secondly, when measuring the development level of inclusive finance, a large number of literatures pay attention to the breadth of coverage and depth of use of financial services [46, 47]. Few literatures have included commercial sustainability in the evaluation index system of inclusive finance development, while commercial sustainability plays an equally important role in the development of inclusive finance. Inclusive finance should seek a balance between commercial profit and social responsibility, which is conducive to the sustainable development of inclusive finance. This paper adds the dimension of commercial sustainability of inclusive finance and finds that it does not have a positive effect on farmers’ income. Finally, from the perspective of functional finance theory, we have studied the strategies and methods of inclusive finance to support farmers’ continuous income growth. In the past, when studying inclusive finance, the scholars focused on its financing functions and ignored its resource allocation functions. This paper demonstrates that inclusive finance can promote the upgrading of the industrial structure by playing the "hematopoietic function" and "guiding role" to increase farmers’ income.

However, there are some limitations that might be addressed by analysis in the future. First, there is room to expand the sample size. Due to the limited availability of data, we only select data at the provincial level and set the sample time interval from 2006 to 2016. We hope to dig deeper into data at municipal and county level on this topic for further research. Secondly, there are relatively few scholars studying the UIS from the perspective of rural areas, and thus there is a lack of sufficient reference basis to measure RIS and AIS more accurately. Future research could build a more perfect RIS and AIS measurement system. Finally, the indicators involved in the inclusive finance index constructed in this paper are based on previous research results, China’s actual situation and the availability of relevant data, and the inclusive finance index system is not perfect enough. Therefore, choosing more reasonable and comprehensive indicators to improve the inclusive finance index system is also an important aspect of future research.

## Supporting information

S1 Data (XLS)

S2 Data (XLS)

S3 Data (XLS)

S4 Data (XLS)
